# Relationship of a new anthropometric index with left ventricular hypertrophy in hypertensive patients among the Han Chinese

**DOI:** 10.1186/s12872-022-02463-6

**Published:** 2022-01-26

**Authors:** Shuang Cai, Jing Dong, Bokai Cheng, Anhang Zhang, Jin Sun, Man Li, Yongkang Su, Qiligeer Bao, Ping Zhu, Shuxia Wang

**Affiliations:** 1 Medical School of Chinese PLA, Beijing, China; 2 Department of Geriatrics, the 2nd Medical Center, Chinese PLA General Hospital, 28 Fuxing Road, Beijing, 100853 China

**Keywords:** A body shape index, Body roundness index, Obesity, Left ventricular hypertrophy

## Abstract

**Background:**

This study aimed to assess the relationship of a new anthropometric index with left ventricular hypertrophy (LVH) in hypertensive patients among the Han Chinese.

**Methods:**

The study is a community-based cross-sectional study that included 4639 patients with hypertension and integrated clinical and echocardiographic data. Left ventricular (LV) mass was measured by transthoracic echocardiography. LVH was diagnosed by using the criteria of left ventricular mass indexed (LVMI) over 49.2 g/m^2.7^ for men and 46.7 g/m^2.7^ for women. Quartiles of a body shape index (ABSI), body roundness index (BRI), waist circumference (WC), and body mass index (BMI) were used regarding LVH prevalence. The logistic regression model was used to determine the odds ratio (OR) and 95% confidence intervals (CI) of the new anthropometric index and LVH. Receiver operating characteristic (ROC) curve analysis was used to evaluate the predictive ability of the obesity indices for LVH risk.

**Results:**

The prevalence of LVH increased across quartiles for ABSI, BRI, BMI, and WC. Comparing the lowest with the highest quartile, adjusted OR (95% CI) for LVH were significantly different for BRI 3.86 (3.12–4.77), BMI 3.54 (2.90–4.31), and WC 2.29 (1.88–2.78). No association was observed for ABSI. According to ROC analysis, the area under the curve (AUC) of BRI was (AUC: 0.653, 95% CI 0.637–0.669), BMI (AUC: 0.628, 95% CI 0.612–0.644), WC (AUC: 0.576, 95% CI 0.559–0.593), ABSI (AUC: 0.499, 95% CI 0.482–0.516).

**Conclusions:**

This study shows that LVH prevalence increased per quartile across the Han Chinese population with hypertension for ABSI, BRI, BMI, and WC. There is a significant association between BRI and LVH in hypertensive people, while ABSI was not. BRI showed potential for use as an alternative obesity measure in the assessment of LVH.

## Introduction

For decades, obesity has been an increasingly serious global health problem in China and the world. Multiple studies have shown that the accumulation of visceral fat, rather than subcutaneous fat, has been identified as being strongly associated with insulin resistance, high blood pressure and dyslipidemia, which increase the risk of cardiovascular disease [1–3]. The incidence of overweight and obesity in adults, as defined by BMI, has risen globally since the 1980s, with no country showing a downward trend [4]. However, people have gradually found that BMI fails to distinguish between the accumulation of muscle and fat, leading to muscular individuals being misdiagnosed as overweight or obese [5–7]. In addition, BMI failed to detect an increase in abdominal obesity, confirming the limitations of using BMI alone to identify obesity phenotypes, which represent the greatest health risk [8]. Although WC is generally considered to be a simple anthropometric indicator of central obesity and related cardiometabolic risks [9, 10], heterogeneity of composition of abdominal tissues and their location-specific in different races do not allow a simple definition of abdominal obesity. Therefore it cannot be used universally across genders or races [11]. As a result, researchers continue to explore new anthropometric indices to better solve the above limitations.

Recently, novel anthropometric indices combining traditional measurement methods (height, weight, WC) have been explored [12–17]. Thus, BRI and ABSI have been suggested as an alternative to traditional anthropometric indices [16, 17]. ABSI introduced by Krakauer et al. is calculated based on height, weight, and WC. ABSI was initially developed to predict mortality hazard in a follow-up study. A high ABSI relates to a greater fraction of abdominal adipose tissue [16]. According to the study of Biolo et al.[18], ABSI is a more direct marker of abdominal adiposity than visceral adiposity. Studies have shown that ABSI is positively associated with fat mass and negatively with fat-free mass [19]. ABSI is positively correlated with visceral adiposity and has been also shown to be positively associated with visceral fat mass [20]. Therefore, ABSI can be used as a practical criterion to predict adiposity-related health risks in clinical assessments [18]. Similarly, in 2013 Thomas et al. have developed the BRI, a geometric index that combines height and WC to predict the percentage of total and regional fat, which allows estimation of the shape of the human body figure as an oval or ellipse. The bigger values of BRI meant that participants had larger amounts of subcutaneous adipose tissue. The values of BRI closer to 1 were related to leaner individuals, and larger values were associated with rounder individuals. Furthermore, as demonstrated by Thomas et al. the advantage of the BRI was that it also could be used to estimate the amount of body fat percentage and gave therefore a better impression of physical health status [17]. Studies have shown that obesity can increase cardiac fat accumulation which is frequently associated with LVH [21]. LVH increases the risk of cardiovascular events and death [22], and reversal of LVH can significantly reduce the risk of cardiovascular events and death [23]. Therefore, early identification of LVH is particularly important. However, data on the relationship between these new obesity indices and the risk of cardiovascular disease are inconsistent [24, 25]. There was no study to assess whether these new anthropometric indices were associated with LVH in a population with hypertension. We conducted this cross-sectional study based on the Han Chinese hypertensive population to assess the relationship of the new anthropometric index with LVH in hypertensive patients among the Han Chinese.

## Methods

### Study population

The details of our research program and exclusion criteria have been described above [26, 27]. Briefly, this community-based cross-sectional study was conducted in Xinyang County, central China, from 2004 to 2005. A multi-stage cluster sampling method was used to select rural community residents aged 40–75 as representative samples. A total of 13,444 subjects (5270 males and 8174 females) completed the survey, and the response rate was 84.9%. Among them, 5421 hypertensive patients were identified and thoroughly examined. Hypertension was defined as diastolic blood pressure (DBP) of ≥ 90 mmHg, systolic blood pressure (SBP) of ≥ 140 mmHg, physician diagnosis, or current hypertension medication (defined by WHO in 1999). Of 5421 hypertensive patients, 4805 patients had measured LVM through echocardiography, and 116 participants were excluded for lack of anthropometric data. Ultimately, 4639 patients with integrated clinical and echocardiographic data were enrolled in the present study. The blood pressure was measured by a well-trained professional with a standard mercury sphygmomanometer. The participants should be at rest for at least five minutes and not consume caffeinated drinks or exercise before the blood pressure measurement. Measurements were taken three times, at least 30 s apart, and three average readings of the sitting posture were taken for analysis.

The study protocol was reviewed and approved by the ethical committees of the Fuwai Hospital and local hospitals. The research procedures followed the ethically normative criteria. All participants provided written consent forms and identified themselves as Han Chinese before being recruited. All investigators were trained at the Cardiovascular Institute, Chinese Academy of Medical Sciences (Beijing, China). After the training course, all participants passed the test.

### Echocardiography measurements

Transthoracic echocardiography was performed according to a standard protocol [28]. The details of the procedures were previously described [27]. The echocardiographic examination was supervised by 2 physician-echocardiographers with at least 2 years of experience. Two technicians from each center performed all the echocardiographic studies. Correct orientation of planes for 2D and Doppler imaging was confirmed using standard procedures [29]. LV internal dimensions and septal and posterior wall thicknesses were measured in up to three cardiac cycles at end-diastole and end-systole according to the American Society of Echocardiography recommendations [28]. A cardiologist read the images without knowing the subjects' clinical characteristics.

LV mass was calculated by using the equation: 0.8 × 1.04[(IVS + LVEDD + PW)^3^ − LVEDD^3^] × 0.6 [30]. IVS was interventricular septum, PW was the posterior wall and LVEDD was left ventricular end-diastolic diameter. LV mass was divided by height^2.7^ to obtain LV mass index (LVMI_h2.7_). LVH was diagnosed using the criteria of LVMI ≥ 49.2 g/m^2.7^ for men and 46.7 g/m^2.7^ for women [31].

### Covariate measurements and definitions

Information about covariates, such as age, gender, and lifestyle, was collected in a single outpatient visit by cardiologists and trained nurses using standard face-to-face interviews. The lifestyle included drinking and smoking. Drinkers were defined as having consumed an average of more than 10 g of pure alcohol per day in the past five years for more than one year. Smokers were defined as individuals who currently smoke an average of one or more cigarettes per day. Ex-smokers were defined as former smokers who had not smoked for three months or more. Participants who were neither current smoker nor ex-smoker were classified as non-smokers [32]. Fasting blood samples were taken from all subjects in the morning (after fasting for at least 12 h). Blood samples were collected from the anterior cubital vein in a vacuum tube containing EDTA. Fasting plasma glucose (FPG), total cholesterol (TC), low-density lipoprotein cholesterol (LDL-C), high-density lipoprotein cholesterol (HDL-C), triglyceride (TG), uric acid (UA), electrolytes, and renal function indicators were analyzed by an automatic analyzer. All laboratory equipment was calibrated, and blinded duplicate samples were used.

### Anthropometric indices assessment

Anthropometric measurements were taken by trained and qualified researchers on subjects wearing light-colored clothes and bare feet. The weight was measured to the nearest 0.1 kg, and the weight was measured to the nearest 0.1 kg. BMI was calculated as weight in kilograms divided by the square of height in meters. We measured the WC of the standing subjects with a piece of soft tape located between the lowest rib and the iliac crest. Height, weight, and WC were used to calculate ABSI and BRI.

ABSI was calculated with the following formula: $\frac{WC}{{BMI^{2/3} Height^{1/2} }}$ [16].

BRI was calculated with the following formula: 364.2–365.5 × $\sqrt {1 - \frac{{(wc{/}(2\pi )^{2} }}{{0.5\;height^{2} }}}$ [17].

### Statistical analyses

Statistical Package for the Social Sciences (SPSS) software version 26 (SPSS Inc., Chicago, IL, USA) was used for data management and statistical analysis. Statistical analyses were initially carried out in the whole hypertension study population (n = 4639), and it was subsequently performed in the population divided into groups based on LVMI over 49.2 g/m^2.7^ for men and 46.7 g/m^2.7^ for women on the presence or absence. Due to the large sample size in this study, we assume that the data are normally distributed. Data were reported as mean ± standard deviation (SD) for continuous variables and as a frequency for categorical variables. Differences in continuous variables between two groups were compared with a *t*-test and differences in categorical variables were measured with the *x*^2^ test. The difference between the LVH group and the non-LVH group was assessed by the independent samples *t*-test.

Pairwise correlation coefficients between the continuous variable height, weight, ABSI, BMI, BRI, and WC were assessed by calculating Pearson correlation coefficients. Quartiles of BRI, ABSI, BMI, and WC were created and the prevalence of LVH was calculated in each quartile. Logistic regression was used to calculate ORs and their 95% CIs. Potential confounders (age, sex, smoking, SBP, TC, UA, creatinine (CR)) were adjusted. ROC curves and AUC were employed to evaluate the predictive ability of the four anthropometric indices for the identification of LVH. Statistical significance was assumed at *P* < 0.05.

## Results

### Clinical characteristics of the study population

There were 1923 cases of LVH among 4639 patients with hypertension. The prevalence of LVH was 41.5%. Table1 shows the clinical and demographic characteristics of the study population by the presence or absence of LVH. Participants of LVH were older than those in the non-LVH group (*P* < 0.001). Male patients comprised 29.5% of the LVH group and 36.6% of the non-LVH group. In the LVH group, there was a relatively high proportion of drinkers (5.4%) and a relatively low proportion of smokers (5.2%). The indicators including age, SBP, DBP, TG, UA, serum urea nitrogen, BMI, WC, and BRI were all higher in the LVH group compared with the non-LVH group, but HDL-C was significantly lower, and no statistical difference was found in ABSI. There were no significant differences in pulse, ALT, AST, K, CL, NA, TC, and CR between the two groups. Moreover, patients with LVH were more frequently women, had a long history of hypertension and higher morbidity of stroke. In terms of echocardiographic parameters, the mean levels of IVST, LVEDD, PWT, RWT, LVM, and LVM/H2.7 were significantly higher among participants with the LVH group.

**Table 1:** Baseline characteristics of study population

Variables	Study population (n = 4639)	LVH (n = 1923)	Non-LVH (n = 2716)	*P*-value
Age (years)	58.0 ± 8.6	59.1 ± 8.3	57.4 ± 8.8	< 0.001
Males (%)	1557 (33.5)	567 (29.5)	990 (36.6)	< 0.001
Smokers (%)	256 (5.5)	99 (5.2)	157 (5.8)	0.001
Drinkers (%)	219 (4.7)	104 (5.4)	115 (4.3)	< 0.001
Pulse (times/min)	72.6 ± 12.3	72.3 ± 12.0	72.9 ± 12.4	0.115
SBP (mmHg)	163.6 ± 24.4	168.7 ± 25.5	160.0 ± 22.9	< 0.001
DBP (mmHg)	97.0 ± 12.5	98.2 ± 13.6	96.3 ± 11.6	< 0.001
ALT (mmol/L)	19.73 ± 13.39	19.31 ± 12.17	20.04 ± 14.18	0.07
AST (mmol/L)	27.90 ± 14.41	27.55 ± 13.35	28.17 ± 15.11	0.154
FPG (mmol/L)	5.57 ± 1.69	5.52 ± 1.65	5.60 ± 1.71	0.114
HDL-C (mmol/L)	1.55 ± 0.34	1.53 ± 0.33	1.57 ± 0.35	< 0.001
LDL-C (mmol/L)	3.15 ± 0.85	3.16 ± 0.87	3.15 ± 0.84	0.562
K (mmol/L)	4.48 ± 0.75	4.47 ± 0.74	4.48 ± 0.77	0.786
CL (mmol/L)	107.50 ± 5.04	107.39 ± 3.84	107.58 ± 5.73	0.214
NA (mmol/L)	143.71 ± 6.02	143.63 ± 4.52	143.77 ± 6.88	0.436
TC (mmol/L)	5.54 ± 1.09	5.53 ± 1.09	5.55 ± 1.10	0.597
TG (mmol/L)	1.69 ± 1.24	1.76 ± 1.31	1.64 ± 1.19	0.001
SUA(μmol/L)	292.85 ± 86.81	295.96 ± 86.12	290.65 ± 87.22	0.044
BUN (mmol/L)	5.47 ± 1.81	5.54 ± 1.85	5.41 ± 1.78	0.016
SCr (μmol/L)	66.26 ± 26.12	66.92 ± 28.86	65.79 ± 24.02	0.155
Stroke (%)	482 (10.6)	247 (13.0)	235 (8.8)	< 0.001
Duration of HTN	7.01 ± 7.61	7.92 ± 8.00	6.28 ± 7.21	< 0.001
*Anthropometric indices*				
BMI (kg/m^2^)	26.20 ± 3.87	27.22 ± 4.17	25.48 ± 3.46	< 0.001
WC (m)	86.11 ± 9.92	87.69 ± 9.93	84.98 ± 9.54	< 0.001
ABSI (m^11/6^ kg^−2/3^)	0.0779 ± 0.0054	0.0779 ± 0.0054	0.0780 ± 0.0054	0.492
BRI	5.20 ± 0.89	5.48 ± 0.89	5.00 ± 0.83	< 0.001
*UCG indices*				
IVST (cm)	1.00 ± 0.16	1.09 ± 0.15	0.93 ± 0.13	< 0.001
LVEDD (cm)	4.55 ± 0.51	4.82 ± 0.47	4.35 ± 0.45	< 0.001
PWT (cm)	0.97 ± 0.14	1.05 ± 0.13	0.91 ± 0.11	< 0.001
RWT	0.43 ± 0.08	0.44 ± 0.08	0.42 ± 0.07	< 0.001
LVM (g)	158.77 ± 44.12	193.36 ± 38.72	134.24 ± 28.53	< 0.001
LVM/H^2.7^ (g/h^2.7^)	46.38 ± 12.48	58.04 ± 9.42	38.11 ± 6.21	< 0.001

SBP, Systolic Blood pressure; DBP, diastolic blood pressure; FPG, fasting plasma glucose; TC, total cholesterol; HDL-C, high-density lipoprotein; LDL-C, low-density lipoprotein cholesterol; TG, triglycerides; SUA, serum uric acid; BUN, serum urea nitrogen; SCr, serum creatinine; ALT, alanine aminotransferase; AST, aspartate aminotransferase; K, Potassium; NA, sodium; CL, chlorine; HTN, hypertension; ABSI, a body shape index; BMI, body mass index; BRI, body roundness index; WC, waist circumference; IVST, interventricular septal thickness; LVEDD, left ventricular end diastolic diameter; LVH, left ventricle hypertrophy; LVM, left ventricular mass; PWT, posterior wall thickness; RWT, relative wall thickness; UCG, ultrasonic cardiogram

### Correlation and prevalence of LVH

ABSI and BRI were positively and significantly correlated to height, weight, BMI, and WC (Table 2). Since indicators such as height and WC were included in BRI and ABSI formulas, a test for multicollinearity was continued. The results showed that they were non-collinearity. The prevalence of LVH increased per quartile for BRI, BMI, WC (1st quartile vs. 4th quartile): BRI 6.3% versus 14.3%, BMI 7.0% versus 13.9%, WC 8.4% versus 11.8% (*P* < 0.01). ABSI showed no significant difference (Table3).

**Table 2:** Correlations between body size and shape

	Height	Weight	ABSI	BRI	WC	BMI
Height	1					
Weight	0.54**	1				
ABSI	0.11**	0.07**	1			
BRI	− 0.52**	0.21**	0.38**	1		
WC	0.23**	0.71**	0.54**	0.59**	1	
BMI	− 0.02	0.72**	0.04**	0.62**	0.72**	1

ABSI, a body shape index; BMI, body mass index; BRI, body roundness index; WC, waist circumference. Correlation coefficients between height, weight, ABSI, BRI, WC and BMI, among the study population (n = 4639)

**Correlation is significant at the 0.01 level (2-tailed)

*Correlation is significant at the 0.05 level (2-tailed)

**Table 3:** Prevalence of LVH in quartiles of ABSI, BRI, BMI, and WC

Quartile	ABSI	BRI	WC	BMI
1 (%LVH [n])	10.2 (475)	6.3 (292)	8.4 (388)	7.0 (323)
2 (%LVH [n])	10.3 (476)	8.9 (415)	10.3 (479)	9.3 (432)
3 (%LVH [n])	10.8 (500)	11.9 (554)	10.8 (502)	11.2 (519)
4 (%LVH [n])	10.1 (471)	14.3 (662)	11.8 (550)	13.9 (645)

ABSI, a body shape index; BMI, body mass index; BRI, body roundness index; WC, waist circumference; LVH, left ventricle hypertrophy

Data presented as mean (SD) or proportion (number)

### Odds ratio (95% CI) of LVH

Table 4 shows the odds ratio (95% CI) of the quartile to LVH (unadjusted and multivariable-adjusted) for each measurement index. Compared with the patients with the lowest quartile of BRI, those with the highest quartile of BRI is significantly associated with the increased LVH risk in the unadjusted model (OR 3.928, 95% CI 3.293–4.685, *P* < 0.001) and after multivariable-adjusted (OR 3.859, 95% CI 3.122–4.771, *P* < 0.001). On the contrary, ABSI did not show any independent association with LVH. BMI and WC were also significantly correlated with LVH (adjusted OR: 3.535 95% CI 2.901–4.307, OR: 2.285 95% CI 1.880–2.777, respectively).

**Table 4:** Binary logistic regression was used to analyze the odds ratio (95% CI) of the quartile to LVH (unadjusted and multivariable-adjusted) for each measurement index

Quartile	ABSI	BRI	WC	BMI	
Unadjusted					
1 (reference)	1	1	1	1	
2	0.996 (0.844–1.175)	1.662 (1.390–1.988)**	1.402 (1.186–1.658)**	1.537 (1.290–1.831)**	
3	1.088 (0.923–1.283)	2.726 (2.287–3.250)**	1.530 (1.294–1.809)**	2.087 (1.756–2.481)**	
4	0.980 (0.831–1.157)	3.928 (3.293–4.685)**	2.114 (1.784–2.505)**	3.245 (2.730–3.858)**	
Multivariate-adjusted					
1 (reference)	1	1	1	1	
2	0.961 (0.803–1.150)	1.647 (1.353–2.004)**	1.451 (1.206–1.746)**	1.698 (1.400–2.057)**	
3	1.028 (0.858–1.231)	2.747 (2.250–3.355)**	1.651 (1.370–1.991)**	2.343 (1.932–2.842)**	
4	0.926 (0.771–1.112)	3.859 (3.122–4.771)**	2.285 (1.880–2.777)**	3.535 (2.901–4.307)**	

ABSI, a body shape index; BMI, body mass index; BRI, body roundness index; WC, waist circumference; LVH, left ventricle hypertrophy. 95% CI, 95% confidence intervals

Adjusted for age, sex, smoking, systolic blood pressure, total cholesterol, uric acid, creatinine. The between cut points are 0.0550, 0.0754 and 0.0779 for ABSI; 4.56, 4.91, 5.42 for BRI; 22.43, 25.30 and 27.64 for BMI; 0.79, 0.86, 0.93 for WC

*Significant at *P* < 0.05

**Significant at *P* < 0.01

We further conducted a stratification analysis of the correlation between LVH and each measurement index category by gender, and the results are shown in Table 5. Regardless of whether female or male, compared with patients in the lowest quartile of BRI, patients in the highest quartile of BRI had a significantly increased risk of LVH with the unadjusted model (Female: OR 4.481, 95% CI (3.485–5.761), *P* < 0.001, Male: OR 3.905, 95% CI (2.579–5.915), *P* < 0.001) and after multivariate adjustment (Female: OR 4.050, 95% CI (3.074–5.337), *P* < 0.001, Male: OR 3.961, 95% CI (2.527–6.209), *P* < 0.001). In contrast, ABSI was not independently associated with LVH in either women or men. BMI and WC are also significantly associated with LVH in both women and men (adjusted female OR: 3.475 95% CI 2.740–4.407, OR: 2.559 95% CI 2.012–3.255; male OR: 4.186 95% CI 2.925–5.990, OR: 2.042 95% CI 1.470–2.837, respectively).

**Table 5:** Binary logistic regression was used to analyze the odds ratio (95% CI) of the quartile to LVH (unadjusted and multivariable-adjusted) for each measurement index by sex stratification

Adjusted OR (95% CI)					Multivariate-adjusted OR (95% CI)				
Quartile	ABSI	BRI	WC	BMI	Quartile	ABSI	BRI	WC	BMI
Female									
1 reference	1	1	1	1	1 reference	1	1	1	1
2	0.992 (0.812–1.213)	1.983 (1.517–2.592) **	1.557 (1.274–1.902) **	1.662 (1.337–2.066) **	2	0.954 (0.768–1.185)	1.841 (1.380–2.455) **	1.682 (1.350–2.097) **	1.798 (1.417–2.282) **
3	1.124 (0.920–1.373)	2.922 (2.259–3.779) **	1.714 (1.396–2.103) **	2.058 (1.662–2.549) **	3	1.032 (0.829–1.284)	2.777 (2.104–3.666) **	1.851 (1.476–2.321) **	2.265 (1.788–2.870) **
4	0.788 (0.580–1.071)	4.481 (3.485–5.761) **	2.483 (2.003–3.078) **	3.106 (2.515–3.835) **	4	0.991 (0.796–1.233)	4.050 (3.074–5.337) **	2.559 (2.012–3.255) **	3.475 (2.740–4.407) **
Male									
1 reference					1 reference				
2	1.049 (0.779–1.412)	1.439 (1.114–1.858) **	0.998 (0.727–1.369)	1.302 (0.966–1.755)	2	0.971 (0.702–1.342)	1.517 (1.145–2.008) **	1.073 (0.759–1.518)	1.618 (1.161–2.255) **
3	1.065 (0.793–1.430)	3.034 (2.286–4.027) **	1.213 (0.905–1.627)	2.051 (1.527–2.754) **	3	0.986 (0.714–1.362)	3.201 (2.338–4.384) **	1.426 (1.029–1.975) *	2.778 (1.991–3.875) **
4	0.788 (0.580–1.071)	3.905 (2.579–5.915) **	1.669 (1.259–2.214) **	3.315 (2.438–4.508) **	4	0.779 (0.554–1.096)	3.961 (2.527–6.209) **	2.042 (1.470–2.837) **	4.186 (2.925–5.990) **

ABSI, a body shape index; BMI, body mass index; BRI, body roundness index; WC, waist circumference; LVH, left ventricle hypertrophy; 95% CI, 95% confidence intervals; OR, odds ratio

Adjusted for age, sex, smoking, systolic blood pressure, total cholesterol, uric acid, creatinine

^*^Significant at *P* < 0.05

^**^Significant at *P* < 0.01

### ROC of each anthropometric index

**Fig. 1:**
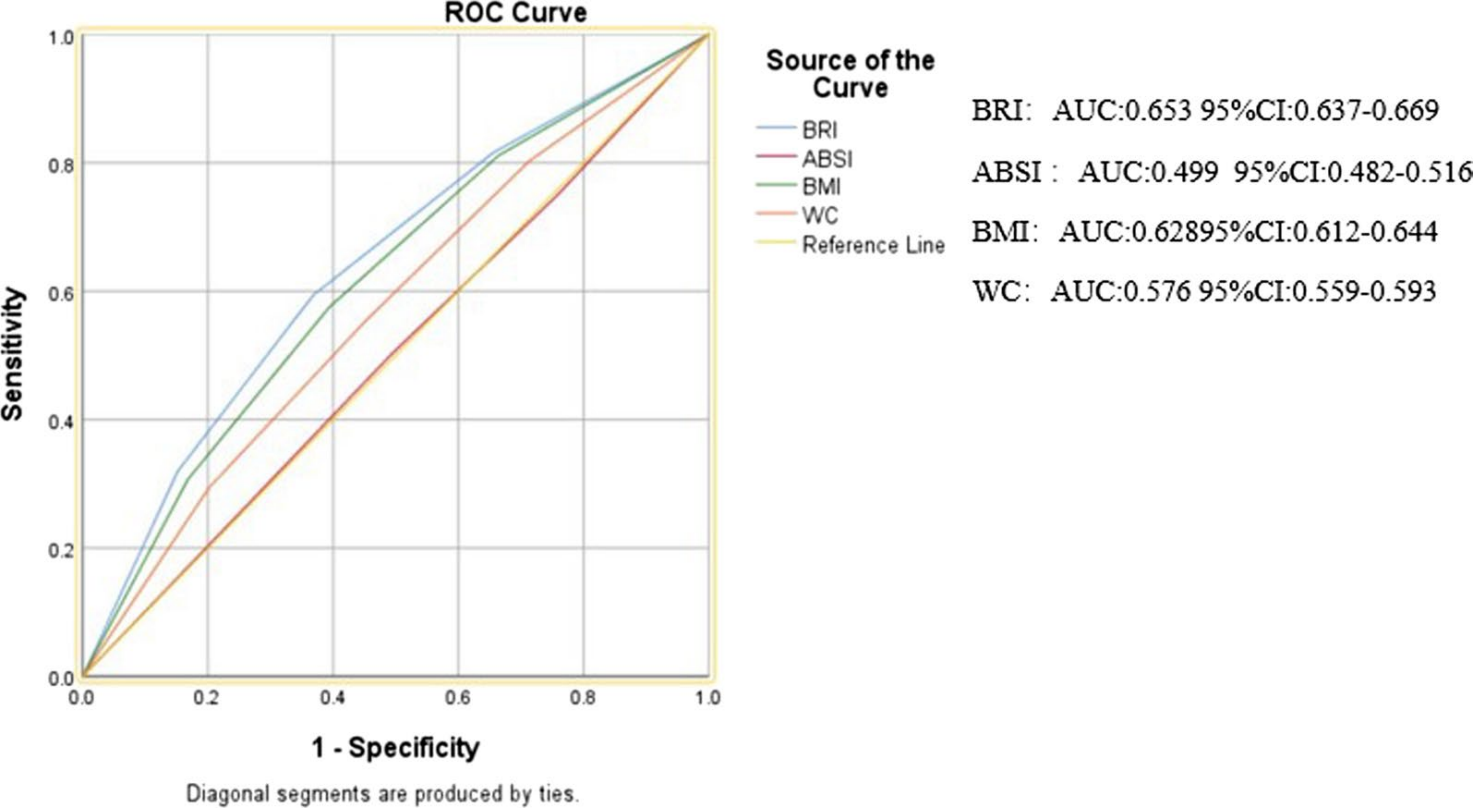
The discriminatory power of ABSI, BRI, BMI, and WC between subjects with or without LVH. ABSI, a body shape index; BMI, body mass index; BRI, body roundness index; WC, waist circumference; LVH, left ventricle hypertrophy; ROC, receiver operating characteristic; AUC, area under the curve

Figure 1 shows the area under the receiver-operating characteristic curve with its 95% CI for identification of LVH by each anthropometric index. According to the ROC analyses, BRI AUC value: 0.653, BMI: 0.628, WC: 0.576, however, the AUC of ABSI had no statistical significances for LVH (AUC < 0.5).

**Fig. 2:**
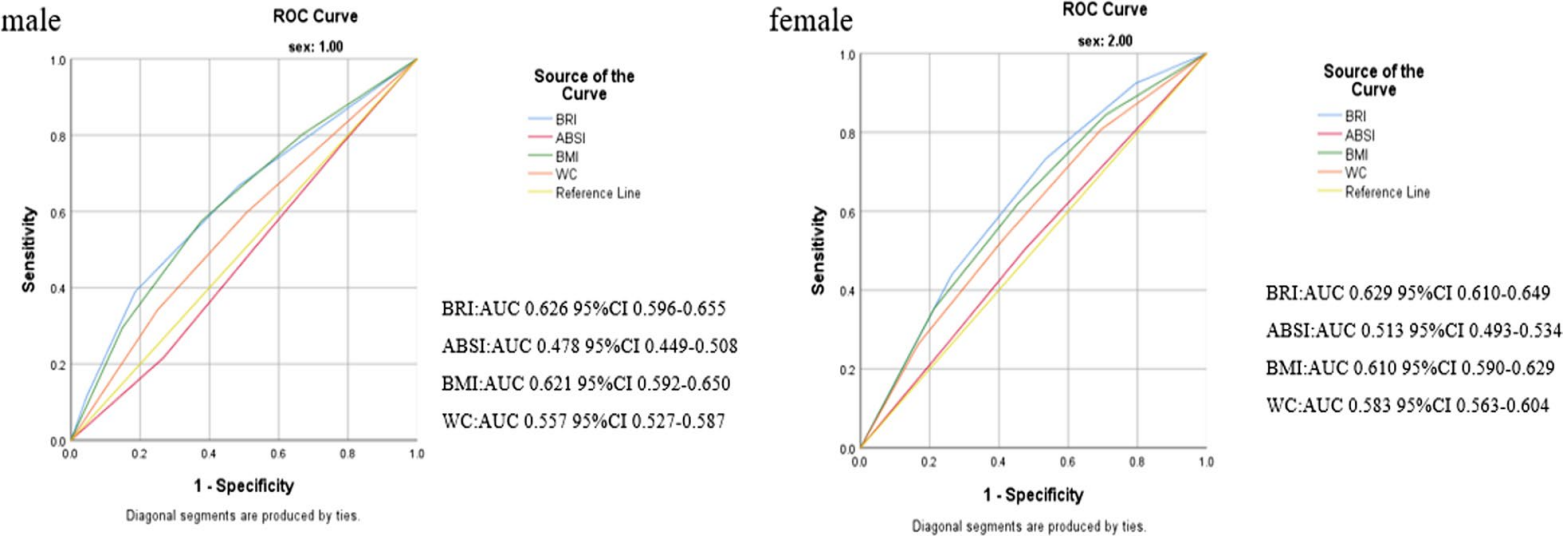
The discriminatory power of ABSI, BRI, BMI, and WC between male and female subjects. ABSI, a body shape index; BMI, body mass index; BRI, body roundness index; WC, waist circumference; ROC, receiver operating characteristic; AUC, area under the curve

Figure 2 shows the AUC for identifying male and female LVH by anthropometric indicators and its 95% CI. As shown in Fig. 2, among men, BRI AUC value: 0.626, BMI: 0.621, WC: 0.557, and in women, BRI AUC 0.629, BMI: 0.610, WC: 0.583. However, the AUC of ABSI had no statistical significances for LVH, whether in men or women (*P* = 0.156, *P* = 0.201 respectively).

## Discussion

This study was conducted in a rural area of Xinyang City, Henan Province, China. In this cross-sectional study, we analyzed the effectiveness of the new obesity index (BRI, ABSI) with other traditional measures in screening LVH risk in the Han Chinese population with hypertension. Our study indicated that the prevalence of LVH significantly increased across the quartiles for ABSI, BRI, BMI, and WC. However, with or without adjustment for potential confounding factors, there is a significant association between BRI and LVH in hypertensive people, while ABSI was not. BMI and WC were also significantly correlated with LVH.

Obesity is a major public health problem worldwide [33]. The prevalence of obesity has gradually increased since the 1980s, with nearly a third of the world's population now classified as overweight or obese [34]. Obesity has many adverse effects on the body, including an increased risk of diabetes, several types of cancers, and cardiovascular disease [35–37]. Obesity patients are often accompanied by increased blood volume, decreased peripheral vascular resistance, and increased heart rate, which together lead to an increase in stroke volume, which ultimately manifests as an increase in cardiac output and volume load. Increased volume and pressure load can further increase the size of myocardial cells, the volume of myocardial cells, change the composition of the collagen matrix, and ultimately cause LVH [38]. LVH is one of the most prominent manifestations of target organ damage in the development of hypertensive patients [39]. It is characterized by ventricular wall thickening, increased myocardial weight, and remodeling of ventricular structure, leading to coronary heart disease, congestive heart failure, and complex ventricular arrhythmias, and is an independent risk factor for cardiovascular events [40]. Obesity is also an important factor causing LVH, and the risks are compounded when both exist together [41, 42]. The mechanism of obesity-related LVH has not been fully elucidated, and it is currently believed that it is mainly caused by hemodynamic factors and neurohumoral factors [38, 43]. The renin–angiotensin–aldosterone system (RAAS), the activation of the sympathetic nerve, hyperleptinemia, obstructive sleep apnea (OSA), and so on all participants in the occurrence of obesity LVH [43]. Different adipose tissues have different effects on metabolic diseases. Visceral fat is the physiologically active component of adipose tissue in vivo. Visceral fat cells have higher lipolysis activity and resistance to insulin's anti-lipolysis effect, and can secrete inflammatory factors acting on blood vessels. With the increase of visceral fat, tumor necrosis factor α, interleukin 6, c-reactive protein and other cytokines increased significantly, which could promote insulin secretion and induce the apoptosis of pancreatic β cells. These factors may participate in the process of left ventricular remodeling hypertrophy, suggesting that visceral fat accumulation is related to LVH.

Despite World Health Organization (WHO) recommends BMI and WC as effective indicators of obesity, and is supported by many studies on its relevance to health risks, the measurement of BMI and WC alone is not enough to help clinicians assess and manage obesity-related health risks in their patients [9]. Some data question the reliability of BMI and WC suggesting that they provide an inappropriate diagnosis of physical obesity [6, 11, 44, 45].

In 2013, Thomas et al.[17] developed a new anthropometric index, BRI, that combines height and WC. BRI reflects both visceral adipose tissue and body fat percentage, which can be used to evaluate health status. BRI has the potential for identifying cardiovascular disease and its risk factors. Zhao et al. showed that BRI was an alternative index for assessing diabetes in Han Chinese people in Northeast China [46]. We demonstrated that BRI was able to identify the presence of LVH in a Han Chinese with hypertension. This is in agreement with previous studies [47]. However, BRI did not show any greater ability to recognize the presence of LVH in the new anthropometric obesity index compared to traditional measures of obesity. As mentioned above, due to the limitations of traditional obesity indicators in defining obesity, we suggest the use of novel obesity indicators to identify obesity and identify obesity-related LVH, although these indicators are more complex to calculate. With the development of computer software accessible via the Internet and smartphone applications, new anthropometric measurements with sophisticated algorithms can be easily used by doctors. Therefore, BRI has the potential to predict LVH more conveniently, more simply, and at a lower cost.

ABSI was created to produce a quantitative measure to estimate the health of body shape. While Krakauer et al. [16] demonstrated that ABSI was a better predictor of premature death than BMI or WC, which was further confirmed in subsequent follow-up studies [48]. He and Chen [49] found that ABSI could be independently used to screen for potential diabetes. However, our results suggest that ABSI cannot be used to distinguish between individuals with and without LVH. Compared with BRI, BMI and WC, there was no significant correlation between ABSI and LVH with or without adjustment for confounding factors. Thus, ABSI is not a suitable measurement method for identifying LVH in the Chinese Han population with hypertension. The underlying mechanism is not clear. The main reason may be due to ethnic differences or subject characteristics [24, 49]. A recent Chinese study of 3,077 community elderly over 65 years of age found that participants with LVH in the highest quartile of ABSI and BRI had a significantly higher risk of LVH than those in the lowest quartile, and both were significantly associated with LVH. Their study also showed BRI was better at predicting LVH in women [50]. Our study showed that ABSI was not associated with LVH and did not appear to have an advantage in identifying LVH, while BRI was a strong predictor of LVH in both men and women. This may be related to the difference in our study population, and it was necessary to conduct larger population studies.

### Limitations

The main strength of the current study was the large sample size of hypertensive patients, which minimizes the selection bias. We estimated the LVM by echocardiographic, which is more sensitive and specific than electrocardiogram. However, there are still some limitations in the current study. First, the gender ratio of males and females was uneven, and more female subjects were recruited in this study. Second, the cross-sectional study design may be unable to distinguish between cause and effect, and follow-up data are needed. Third, gaps in our knowledge remain, and refinement of the anthropometric index for a given across different ages, by sex, and by ethnicity will require further investigation. To address this need, we recommend that prospective studies be carried out in the relevant populations. Finally, since our study took place from 2004 to 2005, hypertension was defined in accordance with the WHO's definition at the time. However, the 2018 clinical guidelines issued by the European Society of Hypertension (ESH) and the European Society of Cardiology (ESC), HTN was defined as an office SBP at least 140 mmHg and/or DBP at least 90 mmHg, which is equivalent to a 24-h ambulatory blood pressure monitoring (ABPM) average of at least 130/80 mmHg, or a home blood pressure monitoring (HBPM) average at least 135/85 mmHg [51]. Since our study was conducted in China, which is in line with the current Chinese HTN management guidelines [52], this is indeed a limitation of this study. However, our results apply to the Han Chinese population.

## Conclusions

In this study, we demonstrated that LVH prevalence increased per quartile across the Han Chinese population with hypertension for ABSI, BRI, BMI, and WC. There is a significant association between BRI and LVH in hypertensive people, while ABSI was not. Given the potential of the new index to identify obesity, we suggest that the BRI may serve as a marker of obesity index to further identify subjects at high risk for LVH in the hypertensive population, especially for obesity-related LVH, thus distinguishing the very high-risk groups that are already in the traditional risk factors.

## Data Availability

The raw/processed data required to reproduce these findings cannot be shared at this time as the data also forms part of an ongoing study. The research data used to support the finding of this study are available from the corresponding authors upon request.

## Abbreviations

ABSI: A body shape index
BMI: Body mass index
BRI: Body roundness index
WC: Waist circumference
SBP: Systolic blood pressure
DBP: Diastolic blood pressure
FPG: Fasting plasma glucose
TC: Total cholesterol
HDL-C: High-density lipoprotein
LDL-C: Low-density lipoprotein cholesterol
IVST: Interventricular septal thickness
LVEDD: Left ventricular end diastolic diameter
LVH: Left ventricle hypertrophy
LVM: Left ventricular mass
PWT: Posterior wall thickness
RWT: Relative wall thickness
UCG: Ultrasonic cardiogram
OR: Odds ratio
95% CI: 95% Confidence intervals
ROC: Receiver operating characteristic
AUC: Area under the curve
WHO: World Health Organization
ESH: European Society of Hypertension
ESC: European Society of Cardiology
ABPM: Ambulatory blood pressure monitoring
HBPM: Home blood pressure monitoring
